# Understanding the Sustainability of Retail Food Recovery

**DOI:** 10.1371/journal.pone.0075530

**Published:** 2013-10-10

**Authors:** Caleb Phillips, Rhonda Hoenigman, Becky Higbee, Tom Reed

**Affiliations:** 1 Department of Computer Science, University of Colorado, Boulder, Colorado, United States of America; 2 School of Medicine, Stanford, Palo Alto, California, United States of America; 3 Community Food Share, Inc., Niwot, Colorado, United States of America; Cinvestav-Merida, Mexico

## Abstract

In this paper we study the simultaneous problems of food waste and hunger in the context of food (waste) rescue and redistribution as a means for mitigating hunger. To this end, we develop an empirical model that can be used in Monte Carlo simulations to study the dynamics of the underlying problem. Our model's parameters are derived from a data set provided by a large food bank and food rescue organization in north central Colorado. We find that food supply is a non-parametric heavy-tailed process that is well modeled with an extreme value peaks over threshold model. Although the underlying process is stochastic, the basic approach of food rescue and redistribution to meet hunger demand appears to be feasible. The ultimate sustainability of this model is intimately tied to the rate at which food expires and hence the ability to preserve and quickly transport and redistribute food. The cost of the redistribution is related to the number and density of participating suppliers. The results show that costs can be reduced (and supply increased) simply by recruiting additional donors to participate. With sufficient funding and manpower, a significant amount of food can be rescued from the waste stream and used to feed the hungry.

## Introduction

There is a contradiction present in the United States (US) today: up to 50% of food produced for consumption is wasted in some stage of production, distribution, or preparation [1]–[3]. Meanwhile, 14.7% of americans (1 in 7) have low food security and and 5.7% have very low food security. A clear question arises when studying these statistics: is it possible to recover food from the waste stream and redistribute it to those who are hungry in a way that reduces both waste and hunger?

The idea of food recovery is not new — there are dozens of non-profit food rescue and gleaning organizations (e.g., [4]–[6]) that have been recovering and redistributing food for more than 30 years. These organizations receive food donations from grocery stores, farms, retailers, and restaurants that are overstock or close to the “best by” date and would otherwise be discarded. Recently, a coalition of major grocers and retailers organized under the Feeding America project with the goal of large scale food rescue, redistribution, and documentation [7]. Yet, to our knowledge there has been no prior effort to quantify the cost and practicality of expanding the current food rescue and redistribution efforts to address hunger on a large scale.

In this paper, we investigate food recovery as a time-sensitive, spatial distribution problem involving food supply and demand and the energy cost of redistribution. Using data provided by a large food bank and food rescue organization in north central Colorado, we build an empirical model for the food waste process, and develop an optimization framework for finding low-cost solutions to food recovery and redistribution. Through simulation we study the average, best case, and worst case bounds on both the amount of food available and recovery costs. By investigating the sensitivity of the model to its parameters, such as food expiry rate and location and density of participating donors, we can also determine the most important components that affect a comprehensive and sustainable food rescue system.

The data we use in this study was supplied by Community Food Share (CFS), the sole food bank for Boulder and Broomfield counties in north central Colorado [8]. This data includes the pounds of food received from 90 distinct donors on each day for one year, July 1, 2010 to August 31, 2011, comprising 20,270 donations and 2,328,821 lbs of food. This food was distributed to 304 unique agencies, which are predominantly homeless shelters, soup kitchens, smaller food pantries, and other organizations that serve at-risk populations.

The goal of our simulations is to reproduce and understand the dynamics of the food recovery problem: how much energy (cost) must be expended to meet the average and worst-case demand, can demand be met reliably, how frequent are underruns (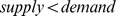) and overages (excess pickup), and how the rate of food expiry limits the amount of recoverable food. We also use the CFS data to extrapolate the supply that could be available from stores in Boulder and Broomfield counties that are not currently donating. In future work, we hope to include restuarants and cafes as well, but have excluded them in the present study because CFS does not pick up from them.

The approach we take for simulation is classic repeated measures, where the average behavior of a complex system is studied through repeated Monte Carlo simulation and *ex post facto* analysis. Each simulation is run for one year (365 days) using the same random seed (so that results can be compared). We use a model parameter, 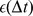 is 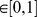, to capture food expiration. An 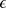 of 0.5, indicates that 50% of “waste” food is expected to expire in 24 hours (or, put another way, half of the food will remain on day 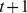, and one quarter on day 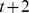, and so on). Using the same set of suppliers, we can evaluate how food availability changes as a function of cost (driving distance) and 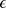.

## Materials and Methods

**Figure 1:**
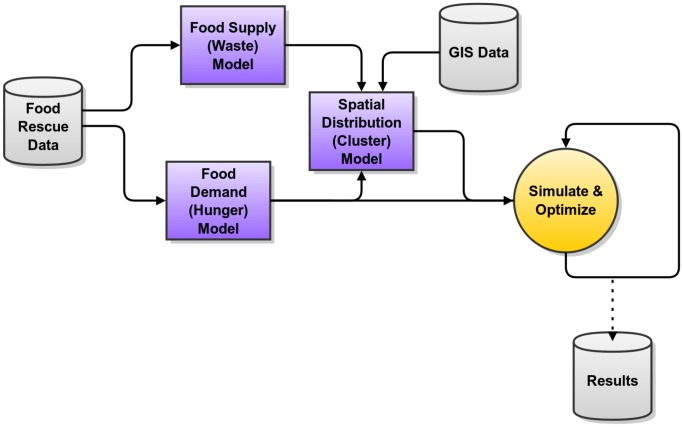
Overview of modeling and simulation process. Food rescue data is used to fit generative statistical models for supply (waste) and demand (hunger). A spatial distribution model uses the supply models and geographic information system (GIS) data to determine the cost of picking up food at donors.

Figure 1 provides an overview of the modeling and simulation process used in this work. First, using the provided data, we build empirical statistical models for food supply (waste) and food demand (hunger). Food supply is a random process where the available recoverable waste at each donor is a function of many factors. However, we find that this quantity can be modeled statistically using a Peaks over Threshold (POT) approach derived from Extreme Value Theory, traditionally used in weather modeling. This model uses the donor category and size (square footage) as inputs. Food demand is less dynamic and is simply a function of the distribution of hunger in the region studied. We find that demand is Gaussian (normally distributed), and use statistics from hunger surveys (e.g., [9]) to derive a set of demand goals.

Next, Geographical Information Systems (GIS) data is used to develop a spatial distribution model. We locate potential donors and compute shortest-path driving routes between all pairs of donors. This information is used to determine which donors are near one another, so that food rescue can be efficiently batched and routed. The driving distance from the central warehousing point to each donor (or cluster of donors) is used as the cost of performing that pick up.

Using the supply, demand, and spatial distribution models, we then simulate the system using a Monte Carlo method. On each iteration (day), an optimal food rescue “schedule” is determined that attempts to meet the demand goal (when possible), and minimize cost (kilometers driven). In order to find the optimal schedule, we formulate the food rescue problem as a linear program, where a food recovery schedule is chosen to minimize cost while meeting demand. This linear program simulates work done by food procurement managers at food banks like CFS to produce a permissable schedule.

Finally, using a repeated measures approach, many successive days are simulated and the emergent behavior can be studied and plotted *ex post facto*. We track the amount of excess food recovered, the prevalence of shortages on days when demand cannot be met, and the cost required on each day. This simulation framework can be used to vary model parameters (i.e., location and storage capacity of warehouse, rate that food expires, etc.) to understand how various choices affect the sustainability (i.e., cost versus value) of the system.

### Statistical Modeling

**Figure 2:**
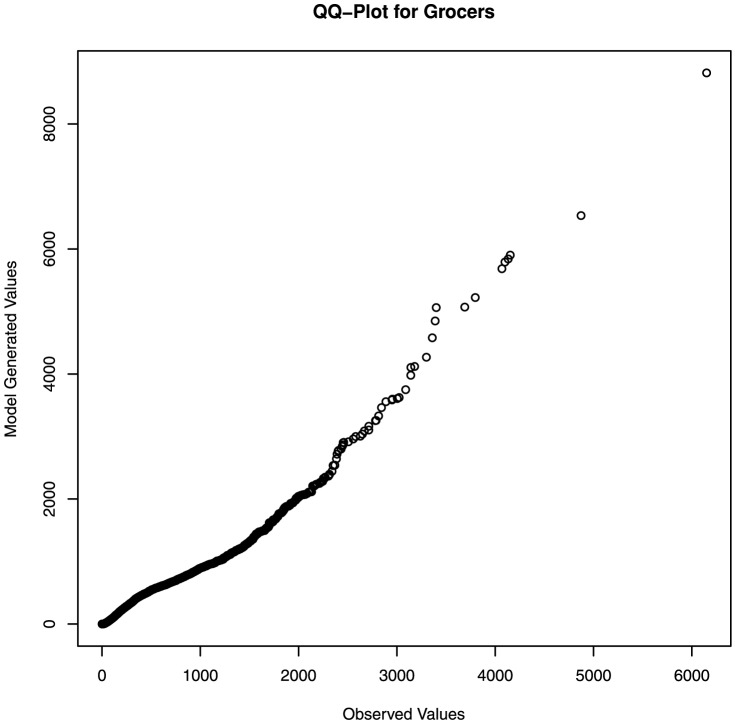
Comparison of observations to model. QQ-plot and histogram comparing observations to model predictions for the ‘Grocers’ category.

We first build a generative statistical model for the food waste available at a given donor as a function of their type (e.g., grocer, manufacturer, or farm), and size. This data has a distinct heavy tail, where distribution is skewed to the left, indicating that small values are most common, but that with small probability, large and sometimes extremely large values can be observed. There is not a clear enough autocorrelation in the data to permit using time-series models. Instead, we find that this process is described extremely well using a peaks over threshold (POT) model where events greater than zero are modeled using a Generalized Pareto distribution with Maximum Likelihood Estimator (MLE) fitted parameters provided for each donor category in table 1. Figure 2 provides a comparison of the fitted model to the observations for the ‘grocers’ category. This model has an illustrative analogy in weather modeling. In Colorado, for example, on many days it does not rain, on some days it rains a little, and on a few days it rains heavily. This pattern corresponds to what we observe in the food rescue data: at a given donor the amount of food that can be rescued is often small, but can occassionally be very large.

**Table 1:** GPD fit parameters for daily supply and demand distributions in pounds. Standard Error values for the fitted parameters are given in parentheses.

Data	Threshold ( 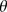 )	Rate ( 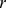 )	Location ( 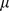 )	Scale ( 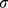 )	Shape ( 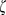 )	Mean
All	0	0.121	0	275.947 (5.382)	0.439 (0.016)	491.884
Grocers	0	0.302	0	293.139 (6.130)	0.205 (0.016)	368.728
Manufacturers	0	0.038	0	562.549 (42.979)	0.107 (0.051)	629.954
Individuals	0	0.029	0	141.755 (18.374)	0.905 (0.126)	1492.042
Farms	0	0.023	0	918.811 (188.314)	0.867 (0.200)	6908.353

The values in table 1 also reveal the donation behavior of each category. Grocers are fairly consistent donors with a rate of 0.302, indicating that a typical grocery store donates some food on about 30% of days, with a mean weight of 369 lbs. Farms, on the other hand, donate infrequently with a rate of 0.023 — they donate on about 2% of days. However, when they donate the mean quantity is much larger than it is for grocers—around 6,000 lbs. Using the fit parameters in table 1, Pareto-distributed daily supply values can be generated for each category:
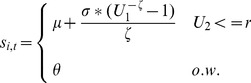
(1)where 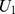 and 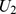 are uniformly distributed random numbers in 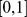. We use this function in our experiments to generate random, correctly distributed supply values that can be used in Monte Carlo-style simulations. Because there is substantially less data available for farms and individual donors, in the remaining analysis we focus on retail donors (grocers, manufacturers, and bakeries). In future work, we hope to leverage other data sources to model the spatiotemporal dynamics of recoverable farm waste.

**Figure 3:**
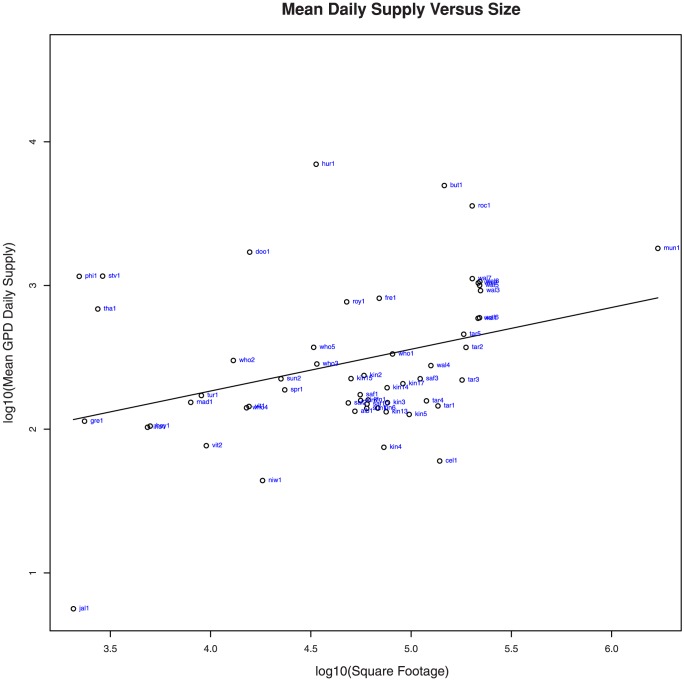
Correlation between donor size and food available. Log/Log-linear correlation between mean daily scale and square footage of participating donors.

The 90 donors included in the CFS data do not represent the complete set of potential donors in the CFS service area. We estimate the list of potential donors in Boulder and Broomfield counties to be 156. In this experiment, farms and individual donors have been excluded, and instead the focus has been an exhaustive set of retail food establishments: grocers, manufacturers, and bakeries. We cannot claim that this list is exhaustive, but we think it captures the bulk of the potential donors. To determine potential supply from these current non-donors, we first use the CFS donor data to correlate other variables with mean daily supply: store size (building square footage), municipal zoning category, and store distance from the CFS warehouse. The result of the ANOVA shows that the most important correlating variables are size and zoning category. The relationship between donor size and mean daily supply appears to follow a power law. An ANOVA gives F-values of 69.042 and 27.841 for 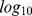 of store square footage and donor category respectively, and 29.548 when used together. The F-value is a statistic that describes the ratio between explained variance and unexplained variance—or, put differently, the ratio of between-group variability to within-group variability. A Pearson's product-moment correlation test confirms this relationship with a statistically significant correlation between the log of donor size and the log of mean daily supply for both grocers alone (p-value 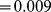 and 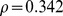), and all suppliers together (p-value 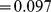 and 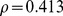). Given this, we can say that the mean daily supply (waste) and variability is independently correlated with the size of the donor. Figure 3 shows this relationship as a scatterplot. This is an important result because it allows us to predict the supply (waste) distribution for a given donor based simply on publicly available information: square footage and municipal zoning category.

A final modeling task is predicting demand. Due to privacy concerns for some agencies, it is not possible to determine the per-agency demand from any defining characteristics, such as agency size or surrounding population density. Instead, we use the aggregate daily demand, which corresponds to the amount of food delivered by CFS or picked up at the warehouse directly by the agencies. During the period for which we have data, CFS distributed food on 294 of 427 days, totaling 4,445,071 lbs. On average, CFS distributes 10,410 lbs of food per day, or 15,119 lbs per day if weekends, holidays, and other closures are excluded. This total distribution amount is approximately twice that donated, since CFS purchases approximately 50% of the food they distribute.

Several reports have detailed food insecurity in the US, reaching different conclusions about the extent of the problem. According to [7], which describes the efforts of the Feeding America program, 5.7 million unique individuals (or 1.661% of the US population in 2009)) are served each week by the approximately 37,000 agencies aligned with their program. There are 348,017 individuals in the service area of CFS, meaning that, based on national-level statistics, there are approximately 5,781 unique individuals in this region per week who need food assistance. In [10], Nord et al. show thatin 2009, 14.7% of households nationally were food insecure at some point during the year, 9% had low food security, and 5.7% had very low food security. Low food security is defined by the USDA as “Reports of reduced quality, variety, or desirability of diet. Little or no indication of reduced food intake” and very low food security is defined as “Reports of multiple indications of disrupted eating patterns and reduced food intake” [11]. Using the 5.7% figure would suggest that 20,490 individuals have very low food security in the CFS service region. A local study conducted by Feeding America in conjunction with CFS found that approximately 10,800 unique individuals seek food assistance per week in the CFS service region [9]. Using USDA statistics for average consumption of food, a typical American in 2010 consumes approximately 2.85 lbs of food per day, of which the majority is meat and protein (0.41 lbs) and grain (0.48 lbs) [12]. This approximation assumes that the weight of dairy products is equivalent to the same volume weight of water and the weight of vegetables and fruit is equivalent to half the weight of the same volume of water. Given this, if we assume that each individual who has very low food security acquires a third of their daily intake via CFS, the mean daily demand would be between 5,491 lbs (using national Feeding America levels), 10,260 lbs (using local CFS Feeding America service statistics, 19,465 lbs (using USDA very low food security percentile), and 48,600 lbs (using USDA low food security perentile). This is a staggeringly large range that serves to highlight the fact that consensus on hunger and food demand does not exist. For the purposes of this study we will focus on the 10,260 number because it is based on carefully collected regional data and agrees with the 10,410 lbs distributed on average observed in the data. Recovering this quantity of food from the waste stream would approximately double the amount currently rescued by CFS, perhaps allowing them to avoid purchasing any food at all.

### Scheduling

The CFS pickup schedule involves visiting a subset of donors each day. Pickups at donors in close proximity to each other are batched together for efficiency. Food is taken to a central warehouse, where it is sorted, weighed, and then re-distributed as needed. Our scheduling strategy searches for a pickup schedule that emulates this hub-and-spoke distribution system. We use a linear program (LP) to find the pickup schedule for each day that meets the demand and minimizes the cost in kilometers traveled. To establish a multi-day schedule, we repeat the linear program for each day. Although this does not guarantee a globally optimal solution, it effectively mimics the problem faced by CFS, where the quantity and location of food cannot typically be known far in advance.

Setting up the linear program starts with 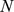 donors with food supply, and 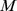 agencies with food demand. The aggregate available food supply (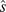) on a given day (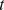) in arbitrary units is the sum of the supply from each individual donor. Similarly, aggregate demand (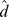) on a given day (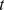) in arbitrary units is the sum of demand at each individual agency.

The multi-day pickup schedule is controlled by a boolean matrix (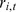), which identifies which suppliers have pickups scheduled on which days:
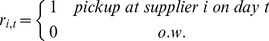
(2)


Each donor is associated with a constant cost of making a pickup (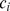). The total cost (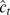) on day (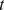) is the cost to visit the selected donors on that day:
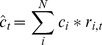
(3)


The total supply for that pickup schedule (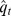) is then:
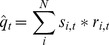
(4)


A multi-day pickup schedule for food presents a unique challenge in that food can go bad. Some of the food not picked up on day 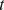 will expire by day 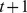, but the other food will remain. We have made the simplifying assumption that all food expires at the same rate regardless of the way the food is stored, state of the food, or type of food. The food supply available on day 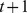 is the new supply for that day, plus the previous day's supply that was not picked up and did not expire:
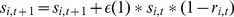
(5)where 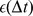 is 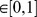 and quantifies the fraction of food not expected to expire over 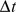 nights. 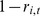 is the logical inverse of the boolean scheduling matrix (and hence is 1 when a pickup does not occur and 0 otherwise). This recurrence can be rewritten as a summation of the previous 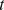 days:
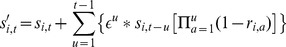
(6)


Another important component to the model is a central warehouse where excess food can be stored after pickup to allow for overages in recovery to be used the following day. The warehouse supply on a given day 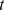 is the amount of food picked up on day 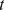 minus the day 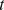 demand plus the warehouse supply from the previous day that did not expire:
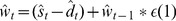
(7)


A sub-optimal schedule for multiple days can be calculated by applying the linear program iteratively for 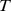 days:
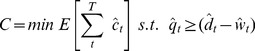
(8)


To calculate distance (cost), we use the MapQuest Driving API directions [13] to retrieve driving directions (which presumably use an optimized shortest path, taking into account the actual constraints of the roads) for each supplier. We use the total driving length of the first offered route, in kilometers, as the cost of visiting that supplier.

Pickups at nearby suppliers are batched using k-means with a radius of 10 km for each cluster. This behavior mimics the scheduling performed by food bank food procurement managers who often build a schedule around the largest and most constrained donors. Although it is possible to compute an optimal route through the selected donors using a combinatorial algorithm (this problem is itself NP-complete, as it is an instance of the classic Traveling Salesman problem), this is extremely computationally costly and would not improve the realism of the simulator. By using a suboptimal route here we have opted for simplicity and realism in design, while erring on the side of conservativity in terms of the sustainability analysis. In future work we hope to explore ways to improve the scheduling systems used by food rescue programs. When a pickup is done at any cluster member, all other cluster members are also visited. The cost of visiting a cluster is the average driving distance between all cluster members and the central warehouse multiplied by two (to count the return trip), plus the minimum sum of the distances between each node. Hence, the cost for visiting a given cluster 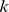 is:
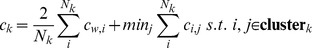
(9)where 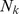 is the number of members in cluster 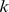, 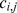 is the cost of driving from 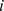 to 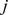, and 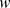 is the index of the warehouse. This cost function replicates the batching of pickups performed by CFS drivers. Although this formulation does not explicitly model all the factors that may contribute to the cost of food recovery, we have chosen to model those key variables that clearly contribute the greatest challenge to the problem, principally: spatial distribution, uncertain (stochastic) supply, and perishability. As we will see, this construction allows the computation of low-cost multiday solutions that provide a reasonable facsimile for those schedules computed by food recovery organizations.

## Results and Discussion

Simulation results show that when we set the daily demand to 5,454 lbs, the mean recieved by CFS in our data set, the demand is met on the majority of days when we use 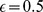. In fact, there is a mean excess of 436.88 lbs a day, indicating that the recovered food from donors is generally higher than the demand. This mean excess value is driven up by spikes of food rescue, which occur at several times during the year. These spikes correspond to extremely large random food rescue events, which are also observed from farms and manufacturers in the real data. This food is recovered for a mean daily cost of 301.72 (kilometers driven). CFS estimates their daily driving distance (a sum of three vehicles, without optimizing paths), to be 212 km. The difference here stems from the fact that our model counts the cost of picking up food at distant farms and manufacturers, which generally deliver the food directly to CFS. If we exclude donors farther away than 100 km, the mean cost drops to 198.35 km, which is within 10% of the value provided by CFS, without producing any additional underruns.

In the next set of simulations, we use a demand goal of 10,260 lbs/day and 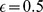, which is the amount that CFS delivers each day. CFS purchases approximately 50% of their food to make up the difference between the mean donations received of 5,454 lbs/day and the mean demand of 10,260 lbs/day. In these simulations, there is a mean shortage of 286 lbs/day, for a mean cost of 1,544 km. If we exclude donors greater than 100 km away, the mean cost drops to 703.1 km, and the average daily shortage also decreases to 262.16 lbs.

There are two takeaways from these results. First, although there is a slight shortage in meeting demand, the amount of food available through food rescue still represents a significant increase over what CFS currently picks up. Next, the observation that in some cases excluding the furthest away donors can substantially reduce cost (by 54% in this case) while obtaining approximately the same amount of food suggests that there may be some fundamental density of donors required for efficient food rescue. In scenarios where donors are sparsely distributed relative to recipients, the cost of rescue may be very high for the same amount of food. On the other hand, in denser environments the model can capitalize on the random supply from nearby (and clustered) donors to drive down cost. Understanding the effect of spatial distribution of donors (and density) is an interesting question for future work.

**Figure 4:**
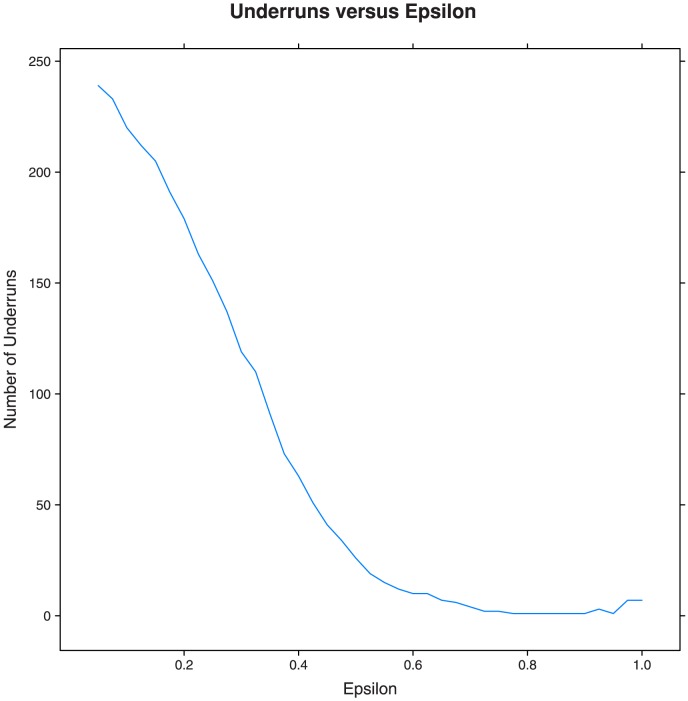
Number of underruns for a 365-day simulation. Number of days when the supply does not meet demand as a function of the epsilon value, using the set of 90 suppliers with a central warehouse.

An important feature of the model is the 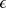 parameter, which controls how quickly food expires. Repeating the 10,250 lbs/day simulations using an 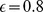, indicating that food expires at a rate of 20%, instead of 50% as above, then demand is met on most days with a mean excess of 182.15 lbs and a mean cost of 215.1 km. This is nearly an eight-fold reduction in cost for the same amount of food, simply by changing the rate at which food expires. Figure 4 shows this relationship explicitly by plotting the number of days in a 365 day simulation where supply failed to meet demand as a function of the 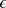 value. For small 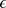 values, the number of underruns is very high; as 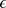 is increased, meeting the demand becomes obtainable and the shortages are mitigated. Clearly, the effect of 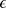 cannot be underestimated. In a practical sense, this highlights the importance of utilizing proper food storage in the recovery process to minimize supply fluctuations and mitigate shortages.

Next, we consider the rest of the suppliers in Boulder and Broomfield Counties that are not currently donating food, but could be, and increase the total set of suppliers from 90 to 156. In this experiment we let 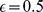 as before. For both the original and this expanded sets of suppliers, there is a saturation point where no additional recoverable food is available. For the set of 90 suppliers this maximum is around 13,000 lbs daily. For the larger set of suppliers, the maximum is closer to 19,000 lbs. There are two conclusions that can be drawn from this result. First, despite the complexity of the underlying model, the relationship between the amount of food available for rescue and the number of donors participating appears to be linear. Second, and perhaps more importantly, with sufficient resources and more participating donors, CFS may be able to comfortably meet their current demand without purchasing food. To meet this goal, they would only need to drive approximately 500 km a day, which is slightly more than two times their current expenditure. This indicates that, provided sufficient funding is available, and a large number of businesses are participating as donors, the food rescue model can successfully feed the area's hungry using only food that would otherwise be wasted. Admittedly, the demand of 10,260 lbs is well below the gold standard of 48,600 based on USDA food security estimates for the area. For that to be met, according to our model, CFS would need to have sufficient resources to drive at least 3,000 km per day.

**Figure 5:**
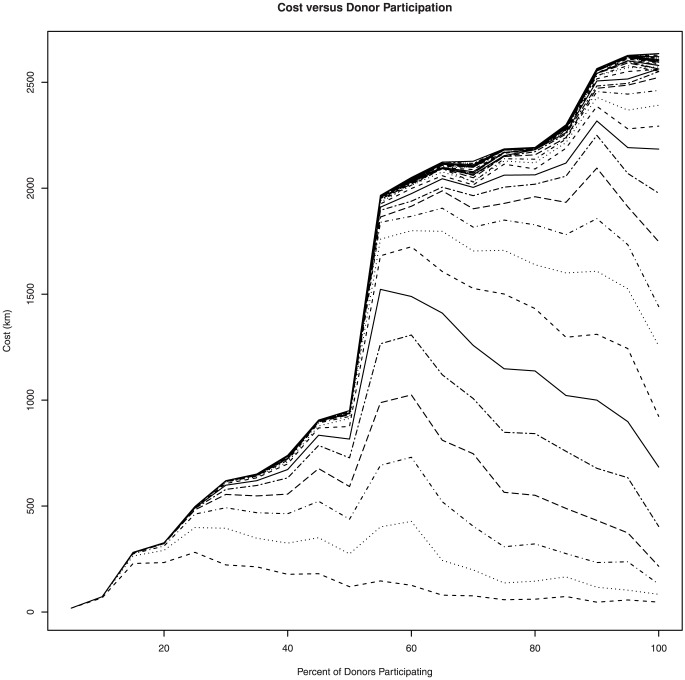
Cost and participating donors. Relationship between cost and percentage of participating donors in the complete supplier set. Each line corresponds to the cost curve associated with a different demand goal between 2,000 lbs (bottom-most line) and 48,500 lbs (line along main diagonal).

Figure 5 shows this relationship between cost and the number of donors explicitly. To generate this graph, we take successively large random samples of the 156 supplier set and run a simulation for a fixed demand goal. In the plot, each line corresponds to the cost required for some fixed demand. Each line exhibits roughly the same behavior: as the number of suppliers increases the cost goes up until a point is reached where the suppliers are able to meet demand (and hence we can optimize solutions to drive down cost). After this point, which is different for each demand goal, the cost required decreases linearly (or superlinearly in some cases) as a function of the fraction of participating suppliers. This indicates that the cost of the food redistribution problem can be reduced simply by increasing the number of participating donors.

## Conclusions

In this paper we provide the first formal investigation of the fundamental sustainabiliy of food recovery. We develop a novel model that can be used for Monte Carlo style simulation using fitted empirical parameters, and we show that this model reproduces the dynamics observed by CFS. While we believe that this model can represent a large class of similar regions with a mix of rural and urban environments, we are careful to remind the reader that our conclusions may not apply in disparite environments (i.e., dense urban or sparse rural). In future work, we hope to integrate additional data in order to broaden our conclusions and perform a more rigorous validation of our model.

Our chief experimental findings in this work are:

- Food supply (waste) events are heavy-tailed and can be well described with extreme value theory “peaks over threshold” models and the generalized Pareto distribution.
- The efficacy of food recovery hinges on the ability to keep rescued food from perishing.
- Despite the underlying heavy-tailed process and complexity of the model, the supply appears to be a linear function of the number of participating donors. Hence, doubling the number of participating donors is likely to double the amount of food available.
- The cost of food recovery can be reduced substantially simply by increasing the number of participating donors (and therefore creating more opportunity for food supply events to occur, when they are required by demand).
- In the scenario we studied in north central Colorado, we have shown that there is substantially more food available than what is currently being recovered. Increasing recovery efforts could reduce both hunger and waste in the region.

In future work, we will expand this investigation to address additional questions. We are interested in whether this model can be scaled up to a state or national level. It stands to reason that dense urban and sparse rural environments will produce substantially different cost and supply dynamics. However, understanding how these dynamics affect the efficacy of food recovery is an open question. There are approximately 85,000 grocery stores and 566,000 food service organizations in the US [14], [15]. In [16], Bloom suggests that the typical food waste associated with a restuarant is on average 3,000 lbs per employee, per year (or 123 lbs/day for a 15 employee restaurant). Clearly, there is no shortage of potential donors; the important question is whether they are well positioned for recovery and whether the cost of rescue is acceptable.

An additional question is one of nutrition. In our current study, we looked at bulk pounds of food without concern for the type. This is a simplification that has bearing on both the economics of the problem (supply and demand) and the basic expiry of the food. Currently, 88% of grocery stores nationally donate some dry goods, 51% donate produce, and 31% donate prepped food and meat [16]. Fresh and healthful foods are the hardest for food banks to acquire since they have a limited shelf life (small 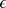), which is negatively affected by transportation, time, stocking time, and pickup limitations (how many pickups per week are possible). Optimizing pickup strategies to capitalize on small food waste events, and sufficiently funding food rescue organizations so that they have the resources to pickup food when it is available might mitigate this problem. A complete solution might require recovery efforts at multiple scales and with varying technologies.

Although preliminary, our work here is an important first step towards understanding the dynamics and limitations of food recovery to mitigate hunger. In the end, we can present the positive result that, despite its underlying complexity, food recovery can be considered a stable process where obtaining additional food is simply a function of having sufficient participating donors and funding to perform pickups. We hope that this work will help to spur interest in this area, equally among researchers who might be able to bring additional insight into the problem, businesses who can agree to donate their food waste, and policy makers who posess the ability to procure needed funding and resources for food rescue organizations to succeed.
